# RLN3/RXFP3 Signaling in the PVN Inhibits Magnocellular Neurons via M-like Current Activation and Contributes to Binge Eating Behavior

**DOI:** 10.1523/JNEUROSCI.2895-19.2020

**Published:** 2020-07-08

**Authors:** Alan Kania, Agata Szlaga, Patryk Sambak, Anna Gugula, Ewa Blasiak, Maria Vittoria Micioni Di Bonaventura, Mohammad Akhter Hossain, Carlo Cifani, Grzegorz Hess, Andrew L. Gundlach, Anna Blasiak

**Affiliations:** ^1^Department of Neurophysiology and Chronobiology, Institute of Zoology and Biomedical Research, Faculty of Biology, Jagiellonian University, Krakow, 30-387, Poland; ^2^Department of Physical Biochemistry, Faculty of Biochemistry, Biophysics and Biotechnology, Jagiellonian University, Krakow, 30-387, Poland; ^3^School of Pharmacy, Pharmacology Unit, University of Camerino, Camerino, 62032, Italy; ^4^Florey Institute of Neuroscience and Mental Health, and Florey Department of Neuroscience and Mental Health, University of Melbourne, Melbourne, 3010 Victoria, Australia

**Keywords:** binge eating, M-like current, paraventricular nucleus of hypothalamus, relaxin-3, RXFP3

## Abstract

Binge-eating disorder is the most common eating disorder. Various neuropeptides play important roles in the regulation of feeding behavior, including relaxin-3 (RLN3), which stimulates food intake in rats through the activation of the relaxin-family peptide-3 receptor (RXFP3). Here we demonstrate that a likely mechanism underlying the orexigenic action of RLN3 is RXFP3-mediated inhibition of oxytocin- and arginine-vasopressin-synthesizing paraventricular nucleus (PVN) magnocellular neurosecretory cells. Moreover, we reveal that, in male and female rats, this action depends on M-like potassium conductance. Notably, higher intra- and peri-PVN RLN3 fiber densities were observed in females, which may constitute an anatomic substrate for observed sex differences in binge-eating disorder. Finally, in a model of binge-eating in female rats, RXFP3 blockade within the PVN prevented binge-eating behavior. These data demonstrate a direct RLN3/RXFP3 action in the PVN of male and female rats, identify the associated ionic mechanisms, and reveal that hypothalamic RLN3/RXFP3 signaling regulates binge-eating behavior.

**SIGNIFICANCE STATEMENT** Binge-eating disorder is the most common eating disorder worldwide, affecting women twice as frequently as men. Various neuropeptides play important roles in the regulation of feeding behavior, including relaxin-3, which acts via the relaxin-family peptide-3 receptor (RXFP3). Using a model of binge-eating, we demonstrated that relaxin-3/RXFP3 signaling in the hypothalamic paraventricular nucleus (PVN) is necessary for the expression of binge-eating behavior in female rats. Moreover, we elucidated the neuronal mechanism of RLN3/RXFP3 signaling in PVN in male and female rats and characterized sex differences in the RLN3 innervation of the PVN. These findings increase our understanding of the brain circuits and neurotransmitters involved in binge-eating disorder pathology and identify RXFP3 as a therapeutic target for binge-like eating disorders.

## Introduction

Relaxin-3 (RLN3) is a neuropeptide expressed mainly in neurons of the brainstem nucleus incertus (NI). Smaller assemblies of RLN3 neurons are scattered within the pontine raphe nucleus (PnR), an area dorsal to the substantia nigra (dSN), and in the medial/ventral periaqueductal gray (PAG). The cognate metabotropic receptor for RLN3, relaxin-family peptide 3 receptor (RXFP3) couples to G_i/o_-proteins and is widely expressed in the CNS (for review, see Bathgate et al., 2013).

RLN3/RXFP3 signaling plays an important role in the control of food intake, arousal and motivation, contextual and spatial memory, neuroendocrine processes, and stress responses (for review, see Ganella et al., 2012, 2013b; Ma and Gundlach, 2015; Blasiak et al., 2017; Calvez et al., 2017; Ma et al., 2017). Moreover, a high sensitivity of RLN3-synthesizing neurons to stress-related factors positions the RLN3/RXFP3 system at the interface between environmental demands and these physiological processes (Tanaka et al., 2005; Ma et al., 2013). The first reported effect of central administration of RLN3 was hyperphagia in satiated, male rats (McGowan et al., 2005). Since then, several studies have further characterized this orexigenic action (McGowan et al., 2005, 2006, 2007; Ganella et al., 2013a; Kania et al., 2017). Moreover, expression of RLN3 mRNA in the NI is elevated in rats with diet-induced obesity (Lenglos et al., 2014). RLN3 peptide signaling is also involved in stress-induced binge-eating (BE), as RLN3 mRNA is elevated in binging female rats, and intracerebroventricular injection of an RXFP3 antagonist prevents the occurrence of binge-like eating (Lenglos et al., 2013; Calvez et al., 2016b). Notably, BE disorder (BED) is the most prevalent eating disorder worldwide (between 0.2% and 4.7% of the adult population), with women affected twice as frequently as men (Kessler et al., 2013). BED is characterized by recurrent BE episodes associated with a sense of lack of control over eating, and is often accompanied by overweight or obesity (American Psychiatric Association, 2013; for review, see Hutson et al., 2018; Novelle and Diéguez, 2018).

Magnocellular neurosecretory cells (MNCs) of the hypothalamic paraventricular nucleus (PVN) have been identified as an important site of the orexinergic action of RLN3 (McGowan et al., 2005, 2006; Ganella et al., 2013a). MNCs in the PVN largely contribute to the pool of oxytocin (OXT) and arginine-vasopressin (AVP) hormones in mammals; peptides involved in a plethora of homeostatic processes, including food intake (for review, see Gimpl and Fahrenholz, 2001; Koshimizu et al., 2012). Among many extrinsic modulatory inputs controlling MNCs, our recent studies described a strong inhibitory influence of RXP3 activation on the activity of PVN MNCs in male Wistar rats (Kania et al., 2017) and revealed that chronic activation of PVN RXFP3 led to increased body weight gain, accompanied by reduced expression of anorexigenic OXT and AVP mRNA (Ganella et al., 2013a).

The physiological roles of both OXT and AVP are sex-specific (de Vries, 2008; Dumais and Veenema, 2016). At the same time, PVN RXFP3 mRNA levels are higher in female than in male rats, and some RLN3 actions are sex-dependent (for review, see Calvez et al., 2017). Intracerebroventricular administration of RLN3 caused stronger hyperphagia in female than in male rats, and when delivered chronically it led to more profound body weight gain in females (Calvez et al., 2015, 2016a; Lenglos et al., 2015).

Despite the well-described role of RLN3/RXFP3 signaling in food intake control, the neuronal and ionic mechanisms underlying RXFP3 action, and its role in stress-related abnormalities in food intake, remain poorly understood. Furthermore, the neuronal basis of the sexual diversity in the influence of RLN3/RXFP3 signaling on food intake is not known. Therefore, we investigated the ionic mechanism underlying the RXFP3-induced inhibition of PVN MNCs, and the influence of RXFP3 activation on MNC synaptic inputs in male and female rats. We also characterized the RLN3 innervation of the PVN area in rats of both sexes. Finally, we assessed PVN RXFP3 signaling involvement in the modulation of BE behavior in female rats.

## Materials and Methods

### 

#### 

##### Ethical approval

All procedures were conducted in accordance with the directive 2010/63/EU of the European Parliament and of the Council of September 22, 2010 on the protection of animals used for scientific purposes. Procedures in immunostaining, neural tract-tracing, patch-clamp, and scRT-PCR experiments were additionally conducted in accordance with the Polish Act on the Protection of Animals Used for Scientific or Educational Purposes of January 15, 2015 and approved by the first and second Local Institutional Animal Care and Use Committee (Krakow, Poland). All efforts were made to minimize suffering and to reduce the number of animals used.

##### Animals and reagents

Male and female Sprague Dawley rats used in immunostaining, neural tract-tracing, patch-clamp, and scRT-PCR experiments were bred and housed in a conventional animal facility. Rats were kept in plastic cages lined with wooden bedding, in constant environmental conditions (21 ± 2°C), maintained on 12-12 light-dark cycle (light on at 08:00 h) with *ad libitum* access to fresh water and standard laboratory rodent chow. From 4 weeks of age, rats were separated from dams and kept in same sex cages, until use in experiments. Rats between 6 and 8 weeks old were used for patch-clamp and scRT-PCR experiments, whereas 3-month-old rats were used for neural tract-tracing studies and 4-month-old rats were used for immunostaining experiments.

For the behavioral experiments, young, female Sprague Dawley rats were purchased from Charles River (Italy) and housed in the animal facility in individual cages for 4 weeks before the experiment and kept in constant environmental conditions (21 ± 2°C), maintained on 12-12 light-dark cycle (light on at 09:00 h) with *ad libitum* access to fresh water and standard food pellets (4RF18).

All reagents for PBS solution, ACSF, and intrapipette solution were purchased from Sigma Millipore, apart from some biocytin batches purchased from Tocris Bioscience (catalog #3349).

Electrophysiology reagent suppliers were as follows: TTX citrate (Abcam, catalog #ab120055 or Tocris Bioscience, catalog #1069), DL-AP5 (Tocris Bioscience, catalog #0105), CNQX (Tocris Bioscience, catalog #1045), bicuculline methiodide (Sigma Millipore, catalog #14343), cadmium chloride hydrate (Sigma Millipore, catalog #529575), tetraethylammonium-chloride (Tocris Bioscience, catalog #3068), 4-aminopiridine (Tocris Bioscience, catalog #0940), and XE991 dihydrochloride (Abcam, catalog #ab120089).

Peptides used were as follows: RLN3 (Phoenix Pharmaceuticals, catalog #035-36A), RXFP3 agonist, RXFP3-A2 {[R3A(11-24,C15→A)B]}, synthesized using solid-phase peptide synthesis and purified using reverse-phase HPLC (Florey Institute of Neuroscience and Mental Health, Victoria, Australia), and the RXFP3 antagonist, R3 (B1-22)R (kindly provided by K.J. Rosengren, University of Queensland). All peptides were dissolved in deionized water, aliquoted, and stored at −20°C, except RLN3, which was dissolved in 10% acetonitrile solution in deionized water and kept in low protein-binding tubes to prevent peptide adsorption to the plastic surface during storage at −20°C.

Immunostaining reagent suppliers were as follows: formaldehyde 36%–38% solution (POCH, catalog #432173111), Triton X-100 (Sigma Millipore, catalog #11332481001), normal donkey serum (NDS, Abcam, catalog #ab7475), avidin-fluorescein conjugate (Sigma Millipore, catalog #94091), mouse anti-OXT antibody (Abcam, catalog #ab78364; or Merck Millipore, catalog #MAB5296), rabbit anti-vasopressin antibody (Abcam, catalog #ab39363), anti-mouse AlexaFluor-647-conjugated antibody (Jackson ImmunoResearch Laboratories, catalog #715-606-150), anti-rabbit Cy3-conjugated antibody (catalog #711-165-152), ImmPRESS HRP anti-mouse IgG polymer detection kit (Vector Laboratories, catalog #MP-7402), DAB peroxidase substrate kit (Vector Laboratories, catalog #MP-4100), and Fluoroshield with DAPI (Sigma Millipore, catalog #F6057). The mouse RLN3 antibody was prepared in-house (Florey Institute of Neuroscience and Mental Health) using a monoclonal cell line originally supplied by the International Patent Organism Depository National Institute of Advanced Industrial Science and Technology (Tsukuba, Ibaraki, Japan). Primary antibodies and NDS were aliquoted and stored at −20°C; secondary antibodies were aliquoted and stored at −80°C.

RT-PCR reagent suppliers were as follows: RNase inhibitor (Thermo Fisher Scientific, catalog #EO0381), Maxima H Minus cDNA Synthesis Master Mix, with dsDNase (Thermo Fisher Scientific, catalog #M1681), dNTPs (Thermo Fisher Scientific, catalog #R0192), primers (Genomed), and Taq DNA polymerase (Thermo Fisher Scientific, catalog #EP0401).

##### Whole-cell recording: tissue preparation, data acquisition and analysis

Whole-cell recordings were performed as described previously (Blasiak et al., 2015; Kastman et al., 2016; Kania et al., 2017). In brief, male and female Sprague Dawley rats (6–8 weeks old) were anesthetized with isoflurane (AErrane, Baxter) and decapitated between 02:00 and 03:00 ZT. Brains were collected in ice-cold, low-sodium, high-magnesium ACSF, containing the following (in mm): 185 sucrose, 25 NaHCO_3_, 3 KCl, 1.2 NaH_2_PO_4_, 2 CaCl_2_, 10 MgSO_4_, and 10 glucose, pH 7.4 (osmolality 290-300 mOsmol kg^−1^) and cut into 250-µm-thick coronal sections on a VT 1000S vibrating microtome (Leica Instruments). Sections containing the PVN were bisected along the third ventricle and transferred to an incubation chamber containing carbogenated, warm (32°C) ACSF, containing the following (in mm): 118 NaCl, 25 NaHCO_3_, 3 KCl, 1.2 NaH_2_PO_4_, 2 CaCl_2_, 1.3 MgSO_4_, and 10 glucose, pH 7.4 (osmolality 290–300 mOsmol kg^−1^). After a recovery period (90-120 min), slices were transferred to a recording chamber, where the tissue was perfused (2 ml min^−1^) with carbogenated, warm (32°C) ACSF of the same composition.

Recording micropipettes were fabricated from borosilicate glass capillaries (5-7 MΩ; Sutter Instruments) using a horizontal puller (Sutter Instruments) and filled with a solution containing the following (in mm): 145 potassium gluconate, 2 MgCl_2_, 4 Na_2_ATP, 0.4 Na_3_GTP, 5 EGTA, 10 HEPES, pH 7.3 (osmolality 290-300 mOsmol kg^−1^) and biocytin (0.05%, for subsequent immunofluorescent identification of recorded neurons). The calculated liquid junction potential for this ACSF and intrapipette solution was 15 mV; and this value was subtracted from the data.

PVN neurons were localized and approached using an Axio Scope FS2 and Examiner D1 microscope (Carl Zeiss) equipped with video-enhanced infrared differential interference contrast. Cell-attached and whole-cell configurations were obtained under visual control using a negative pressure delivered by an ez-gSEAL pressure controller (NeoBiosystem) or mouth suction. SEC 05LX amplifiers (NPI), Micro 1401 mk II (Cambridge Electronic Design) converters and Signal and Spike2 software (Cambridge Electronic Design) were used for signal recording and data acquisition. Recorded signal was low-pass filtered at 3 kHz and digitized at 20 kHz.

PVN neurons were identified based on their unique electrophysiological properties. Type I neurons (putative MNCs) were identified by the presence of the transient outward rectification underlined by robust A-type potassium current (Luther and Tasker, 2000). Activation of this current results in a delay to the first action potential when an MNC is depolarized from the hyperpolarized membrane state (−100 mV). Type II cells (putative parvocellular neurons) lack the delay and display low-threshold spikes caused by T-type calcium current (Luther and Tasker, 2000). Only Type I neurons were the subject of the present study, and only those with stable input resistance throughout recording, monitored based on the voltage or current responses to hyperpolarizing current or voltage steps (applied every 30 or 60 s), were included in the final analysis. According to these criteria, 244 MNCs (123 from male and 121 from female brains) recorded in the slices obtained from 117 young, adult Sprague Dawley rats (59 males and 58 females) were included in the current study. Only one neuron per slice was subjected to peptide administration in these experiments. All peptides and drugs were applied via bath perfusion system.

Current-clamp recordings (zero holding current) aimed at investigating MNC sensitivity to RXFP3 activation were performed in normal ACSF followed by ACSF containing TTX (0.5 μm, to block action potential generation) and antagonists of ionotropic glutamate and GABA receptors (10 μm CNQX, 50 μm DL-AP5, and 20 μm bicuculline, respectively). Voltage-clamp recordings (command potential −50 mV) aimed at investigating MNC sensitivity to RXFP3 activation and the ionic mechanism of RXFP3-mediated inhibition were performed in ACSF containing TTX, CNQX, DL-AP5, and bicuculline and additional compounds: Cd^2+^ (200 μm), tetraethylammonium-chloride (10 mm), 4-aminopiridine (100 μm), and XE991 (10 μm and 50 μm). In a subset of experiments, ACSF with increased [K^+^] (final concentration: 21.6 mm, with a simultaneous decrease in NaCl concentration) was used. Voltage-clamp recordings (command potential −50 mV) aimed at investigating the influence of RXFP3 activation on spontaneous and miniature synaptic currents were performed in normal ACSF and ACSF-containing TTX, respectively. Calculated reversal potential for Cl^−^ currents in our patch-clamp recordings equaled −90.51 mV; therefore, at the given command potential, outward events represented IPSCs, whereas inward events represented EPSCs. The nature of postsynaptic currents was verified using glutamate (CNQX 10 μm, DL-AP5 50 μm) and GABA (bicuculline 20 μm) receptor antagonists.

After recording, slices underwent immunofluorescent staining to examine neurochemical content of recorded neurons. The procedure was conducted according to described protocols (Blasiak et al., 2015; Kastman et al., 2016; Kania et al., 2017). Briefly, slices were fixed overnight with 4% formaldehyde at 4°C. Fixed free-floating sections were blocked and permeabilized (10% NDS, 0.6% Triton X-100 in PBS) at 4°C overnight and incubated with a primary antibody solution as follows: mouse anti-OXT (1:10 000 or 1:5000 depending on the antibody batch), rabbit anti-vasopressin (1:500 or 5000 depending on the antibody batch), avidin-fluorescein conjugate (1:200), 2% NDS, 0.3% Triton X-100 in PBS, for 72 h at 4°C. Following incubation with secondary antibodies: donkey anti-mouse AlexaFluor-647 (1:400), donkey anti-rabbit Cy3 (1:400), 2% NDS in PBS for 24 h at 4°C, sections were coverslipped with Fluoroshield containing DAPI and imaged with a fluorescence microscope (Axio Imager M2, Carl Zeiss).

##### Single-cell RT-PCR (sc-RT-PCR)

PVN-containing brains sections from 6 Sprague Dawley rats (3 males and 3 females) were prepared similarly to the protocol characterized above, and MNCs were recorded using described whole-cell protocols with 1-2 MΩ recording micropipettes. After electrophysiological verification of the cell type, MNCs were voltage-clamped at −50 mV, and the cytoplasmic content was aspired into the recording micropipette while whole-cell current was being monitored. The aspiration lasted until the interruption of the whole-cell configuration. The cytoplasmic samples were deposited in plastic tubes containing 0.5 µl of RNase inhibitor and stored at −80°C until the PCR was conducted to investigate the expression of the following mRNA species: RXFP3, KCNQ2-5, and GAPDH.

DNase treatment and reverse transcription were performed using Maxima H Minus cDNA Synthesis Master Mix, with dsDNase (Thermo Fisher Scientific) according to the manufacturer's instructions. After reverse transcription, the cDNA for GAPDH, RXFP3, and KCNQ2-5 was amplified in a seminested multiplex PCR. First-round PCR amplification was performed in a 50 μl volume containing 10 mm Tris-HCl, pH 8.8, 50 mm KCl, 0.08% (v/v) Nonident P40, 2 mm MgCl_2_, 250 μm dNTPs, 40 nm primers, 6 μl cDNA, and 1.25 U Taq DNA polymerase. Second-round PCRs were performed separately for each gene in a 25 μl volume; 4 μl of the first-round PCR product was used and the concentration of primers was increased by 10. Detailed sequences of primers used in PCRs are summarized in Table 1. All PCRs were run in a T100TM Thermal Cycler (Bio-Rad) for 34 cycles of 30 s denaturation at 95°C, 1 min annealing at 52°C and 90 s elongation at 72°C, preceded with 3 min denaturation at 95°C and followed by a final elongation period of 5 min at 72°C; 25 μl of second-round PCR products was separated on Midori Green (Nippon Genetics) stained 2% agarose gels and visualized by UV trans-illumination. Predicted sizes of the PCR amplicons were as follows: GAPDH, 284 bp; RXFP3, 422 bp; KCNQ2, 216 bp; KCNQ3, 253 bp; KCNQ4, 322 bp; and KCNQ5, 484 bp. All experiments included a control tube, in which RevertAid Reverse Transcriptase was omitted in the reverse transcription negative test.

**Table 1.** Primer sequences used for scRT-PCR experiments*^a^*

	Gene	Pairs of primers (from 5′ to 3′)		
		GenBank no.	First-round PCR	Second-round PCR
GAPDH	AF106860.2	Sense	CCTGCACCACCAACTGCTTAGC	CCTGCACCACCAACTGCTTAGC
		Antisense	CTCGGCCGCCTGCTTCAC	ATGTCAGATCCACAACGGATACATTGG
RXFP3	NM_001008310.1	Sense	CAAGCTCCTGGGTTGGGACC	CAAGCTCCTGGGTTGGGACC
		Antisense	GCATTAAGTGGCGCCAGGGC	GGCGCACTAAGCAGTAGAGGAT
KCNQ2	NM_133322.1	Sense	GTGCTGATTGCCTCCATTGCTG	GTGCTGATTGCCTCCATTGCTG
		Antisense	CCAATGGTTGTCAGGGTGATCAG	GGCCAGGATGAGGCAGAGG
KCNQ3	NM_031597.4	Sense	CTATTCGGACCACATCTCACCC	CTATTCGGACCACATCTCACCC
		Antisense	ACTACAGTACCAGGAGTCAGCC	CATAGGCATGGATCCACTGGG
KCNQ4	XM_233477.8	Sense	GACGATTACACTGACGACCATTGG	GACGATTACACTGACGACCATTGG
		Antisense	CCAGGGCAGAAGGAGGCAC	CTCAAACAAGAGGGCCAGCTC
KCNQ5	XM_001071249.3	Sense	GCTGGGCTCCGTGGTTTACG	GCTGGGCTCCGTGGTTTACG
		Antisense	CTTCTGCACTTTGGTGGGGCTG	GCTTGCTGCCTCCCCTTGTTC

*^a^*GenBank accession number and sequences of primers used for each of the examined genes in both rounds of PCR.

##### Neural tract-tracing: surgeries, tissue preparation, and immunostaining

Injections of neuronal tracer were performed according to described procedures (Blasiak et al., 2015; Kania et al., 2017). In brief, male (*n* = 5) and female (*n* = 8) Sprague Dawley rats (10 weeks old) were anesthetized with isoflurane in an induction chamber, mounted into a small animal stereotaxic system (SAS-4100; ASI Instruments) with a rat gas anesthesia mask (Stoelting) and maintained under deep anesthesia with 3% isoflurane (v/v mixture with air). Retrograde tracers (FluoroGreen, FluoroPink, 40 nl, Tombow Pencil) were injected bilaterally into the PVN (stereotaxic coordinates: AP −1.7 mm [males] −1.5 mm [females], ML 0.4 mm and DV −7.8 mm from bregma) (Paxinos and Watson, 2007), using glass microinjection pipettes (50 µm tip), pulled from borosilicate glass capillaries on a vertical puller (Narishige) connected to 1 µl Hamilton syringe (Hamilton). After 9 d of recovery, rats were again anesthetized with isoflurane for colchicine injection to improve the detection of somatic RLN3 immunoreactivity. Colchicine solution (0.5 mg in 5 µl of 0.9% NaCl made on the day of injection, Sigma Millipore, catalog #C9754) was injected unilaterally into the lateral cerebral ventricle (stereotaxic coordinates: AP −0.7 mm, ML 1.8 mm, and DV −4.0 mm from bregma) using glass microinjection pipettes. After 24 h, rats were anesthetized with pentobarbital (240 mg/kg, i.p., Morbital, Biowet) and killed by transcardial perfusion with 200 ml of saline followed by 200 ml of 4% formaldehyde in PBS (made fresh from 38% solution). Brains were postfixed in 4% formaldehyde in PBS overnight at 4°C and cut into coronal sections on a VT1000S vibrating microtome (Leica Instruments). Brain sections (100 µm thick) containing injection sites were mounted, coverslipped using glycerin, and imaged with a fluorescent microscope (Axio Imager M2, Carl Zeiss). Injection sites were verified and reconstructed using CorelDraw software according to the rat brain atlas (Paxinos and Watson, 2007).

Every third section (50 µm) containing brain regions synthesizing RLN3 (PAG, dSN, PnR, and NI) was immunostained to visualize RLN3-synthesizing neurons according to described protocols (Kania et al., 2017). Briefly, free-floating sections were incubated with 10% NDS, 0.3% Triton-X 100 in PBS for 1 h at room temperature. After blocking and permeabilization slices were incubated with a primary antibody solution (mouse anti-RLN3, 1:50, 2% NDS, 0.3% Triton X-100 in PBS for 72 h at 4°C) and subsequently with a secondary antibody solution (donkey anti-mouse AlexaFluor-647, 1:400, 2% NDS in PBS for 24 h at 4°C). Finally, sections were mounted and coverslipped with Fluoroshield-containing DAPI, and imaged with a fluorescence microscope (Axio Imager M2, Carl Zeiss) and LSM710 confocal laser microscope on Axio Observer Z1 (Carl Zeiss). RLN3-immunoreactive (-ir) neurons containing FluoroPink or FluoroGreen were counted using ZEN 2.1 software and multiplied (×3) to estimate the total cell number.

##### RLN3 fiber immunostaining: perfusion and fixed tissue preparation

In a separate set of immunostaining experiments, male (*n* = 4) and female (*n* = 8) Sprague Dawley rats (4 months old) were anesthetized with pentobarbital (240 mg/kg, i.p.), killed by transcardial perfusion, and their brains were processed as described above (see Neural tract-tracing: surgeries, tissue preparation, and immunostaining).

For visualization of RLN3-positive fibers in the hypothalamus, every third 40 µm coronal section containing PVN was slide-mounted and underwent immunohistochemical staining using a Vector ImmPRESS kit according to the manufacturer's protocol. All procedures were conducted in a humidity chamber on slide-mounted sections at room temperature. Briefly, after blocking the endogenous peroxidase activity (0.3% H_2_O_2_ in PBS for 10 min), sections were permeabilized (0.025% Triton X-100 in PBS for 2 × 5 min) and blocked (2.5% normal horse serum for 20 min). Subsequently, sections were incubated with a primary antibody solution (mouse anti-RLN3, 1:50, 0.3% Triton X-100 in PBS for 90 min) followed by anti-mouse secondary antibody conjugated with HRP polymer (for 30 min). Finally, sections were incubated with a peroxidase substrate solution (DAB for 10 min), rinsed in tap water and coverslipped with glycerin. Stained sections were imaged using a light microscope (Axio Imager M2, Carl Zeiss) with a 20×/0.5 Plan Neofluar objective and an AxioCam MR R3 digital camera.

##### Binge eating experiments: cannula implantation and behavioral testing

Female Sprague Dawley rats (*n* = 20, ∼260 *g* at the beginning of the study) were subjected to bilateral intracranial cannulation surgery, to allow intra-PVN microinjections. Rats were anesthetized with an intramuscular injection of tiletamine chlorahydrate (200 mg/kg) and zolazepam chlorahydrate (200 mg/kg; Virbac), together with a subcutaneous injection of anti-inflammatory meloxicam (1.5 mg/kg) and mounted into a small animal stereotaxic instrument (model 902; David Kopf Instruments). Stainless-steel cannulae (22 gauge; Unimed) were implanted bilaterally and mounted to the skull with stainless-steel screws and dental cement, using the following stereotaxic coordinates: AP −1.6 mm, ML ±1.8 mm, α 10°, DV −7.0 mm from bregma (Paxinos and Watson, 2007). A stainless-steel dummy cannula was placed inside the guide cannula at the end of the surgery. After 14 d of postsurgical recovery, the BE model procedure began.

Microinjections were made using stainless-steel injectors (Unimed) connected with polyethylene-10 tubing to the 10 µl Hamilton syringes (tips of injectors were protruding from the guide cannulae by 1.5 mm). The selective RXFP3 antagonist, R3(B1-22)R was diluted (1 µg/0.5 µl) in ACSF (in mm as follows: 147 NaCl, 4 KCl, 0.85 MgCl_2_, 2.3 CaCl_2_); 0.5 µl of vehicle (ACSF) or R3(B1-22)R solution was manually injected into each side of the PVN (∼0.5 µl/min). The injectors were left in the guide cannulae for ∼30 s after the infusion.

A BE protocol was conducted as described previously (Cifani et al., 2009; Micioni Di Bonaventura et al., 2012, 2013; Pucci et al., 2016; Alboni et al., 2017). Briefly, rats were divided into two weight-matched groups: one exposed to three consecutive 8-d-long cycles of caloric restriction and refeeding (access to 66% of normally consumed chow, days 1-4 of each cycle, followed by *ad libitum* access to chow, days 5–8 of each cycle); while at the same time, a second group of rats with *ad libitum* access to chow throughout the entire period (24 d). Additionally, on days 5–6 and 13–14 (four exposures), rats from both groups were given access to highly palatable food (HPF) for 2 h during the light phase (starting 2 h after the light onset). The HPF (3.63 kcal/g) was a mix of 52% Nutella (Ferrero) chocolate cream (5.33 kcal/g; 56%, 31%, and 7% from carbohydrate, fat, and protein, respectively), 33% grounded food pellets (standard rat chow, Mucedola, 4RF18), and 15% water (w/w percent ratio). On the first BE test day (25th day of the study), rats from the restricted group were exposed to frustration stress. For 15 min, the HPF was placed inside a metallic grid container hanging on the anterior wall of the cage, so that the rats were able to see and smell the HPF but were not able to eat it (frustration stress). Subsequently, the HPF was placed inside the cage and rats started to eat. Rats in the nonrestricted group had immediate access to the HPF, without frustration stress; therefore, the final profile of the experimental groups was restricted and stressed (binge-eaters [BE]), and nonrestricted and nonstressed (control). During the test, the HPF intake was measured over 2 h in several discrete time points (starting ∼2 h after the light onset). Three days after the first BE test, a fourth 8-d-long cycle of food restriction and refeeding was started in the BE group, and constant *ad libitum* access to chow was maintained in the control group. Subsequently, the second BE test (37th day of the study) was conducted (for the protocol timeline, see Fig. 5*A*). On BE test days, bilateral intra-PVN injection were made immediately before the frustration stress (BE group) or 15 min before access to HPF (control group). Before each test, half the rats was injected with R3(B1-22)R and half with vehicle; these conditions were switched between the first and second test, so each rat received both (R3(B1-22)R and vehicle) in separate tests.

After the experiment, brains were collected, frozen in ≤−40°C isopentane, and stored at ≤−80°C until being cut on a cryostat into coronal 40 µm sections to verify the correct bilateral PVN targeting. The sections were examined under a light microscope (Leica DMR, Leica Microsystems) equipped with a camera (DS-Ri2, Nikon Instruments). Based on cannulae placement, 2 rats were excluded from the final analysis of HPF intake (1 control, 1 BE).

##### Experimental design and statistical analysis

Electrophysiological data were analyzed using custom Spike2 and MATLAB (The MathWorks) scripts as well as Mini Analysis software (Synaptosoft) in the case of postsynaptic current recordings. The change in membrane potential or whole-cell current in response to the peptide application was considered significant if it differed from the baseline by >3 SDs. The I--V relationship of RXFP3-A2-induced whole-cell current and its blockade by XE991 was statistically analyzed in a group of MNCs responsive to RXFP3-A2 at a command potential of −50 mV. For postsynaptic current analysis, two 200 s whole-cell recordings (one from the baseline period before drug application, and another 300 s later) were analyzed using Mini Analysis software. Events were manually detected to measure frequency, mean rise time, mean amplitude, and decay time constant of averaged current trace; these parameters were subjected to statistical analysis (baseline vs peptide application). Analysis of postsynaptic currents was restricted to MNCs in which drug application induced an outward whole-cell current. Repeated-measures two-way ANOVA, paired *t* test, unpaired *t* test with Welch's correction, and Fisher's exact test were used for statistical comparisons; and the application of each specific test is indicated in Results.

In the scRT-PCR experiments, only neurons with detectable GAPDH expression were included in the final analysis. A χ^2^ test was used to compare the proportion of male and female neurons expressing the detected mRNA species.

The distribution of RLN3-ir fibers in the PVN area was reconstructed based on *z*-stack images (10 optical slices, with a 1.480 µm step size, 1388 × 1040 pixels) combined into projections and stitched using ImageJ (Schneider et al., 2012) and CorelDraw software, respectively. Images corresponding to the selected plates of a rat brain atlas (distance from bregma: from −1.92 to −1.56 mm) (Paxinos and Watson, 2007) were further processed using a local thresholding method (Bernsen, radius = 1 pixel, contrast threshold = 25) and an Analyze Particles option (size = 0-300 pixel) to dissect the DAB-stained RLN3-ir fibers. Area fraction was measured in defined bilateral ROIs, including the PVN area and adjacent dorsal, lateral, and ventral regions, to determine the density of stained fibers (see Figs. 5*A*, 4*A*). The distribution of fibers was compared between ROIs and sexes using a repeated-measures two-way ANOVA.

The HPF intake during the BE test was expressed as kcal/kg of body weight and compared between groups (control vs BE) and treatments (ACSF vs R3(B1-22)R), using a two-way ANOVA.

The test used is indicated in the Results. Data were considered to be significantly different at *p* < 0.05. All tests were two-tailed. All values are presented as mean ± SD. Statistical analysis was performed using GraphPad Prism version 6.00 for Windows (GraphPad Software).

## Results

### RLN3/RXFP3 signaling inhibits a majority of PVN MNCs in male and female rats

In a series of whole-cell recordings *ex vivo*, we investigated the effects of RLN3 and a selective, high-affinity RXFP3 agonist peptide on the electrophysiology of PVN MNCs in male and female Sprague Dawley rats, and explored possible sex differences in recorded responses.

**Figure 1.**
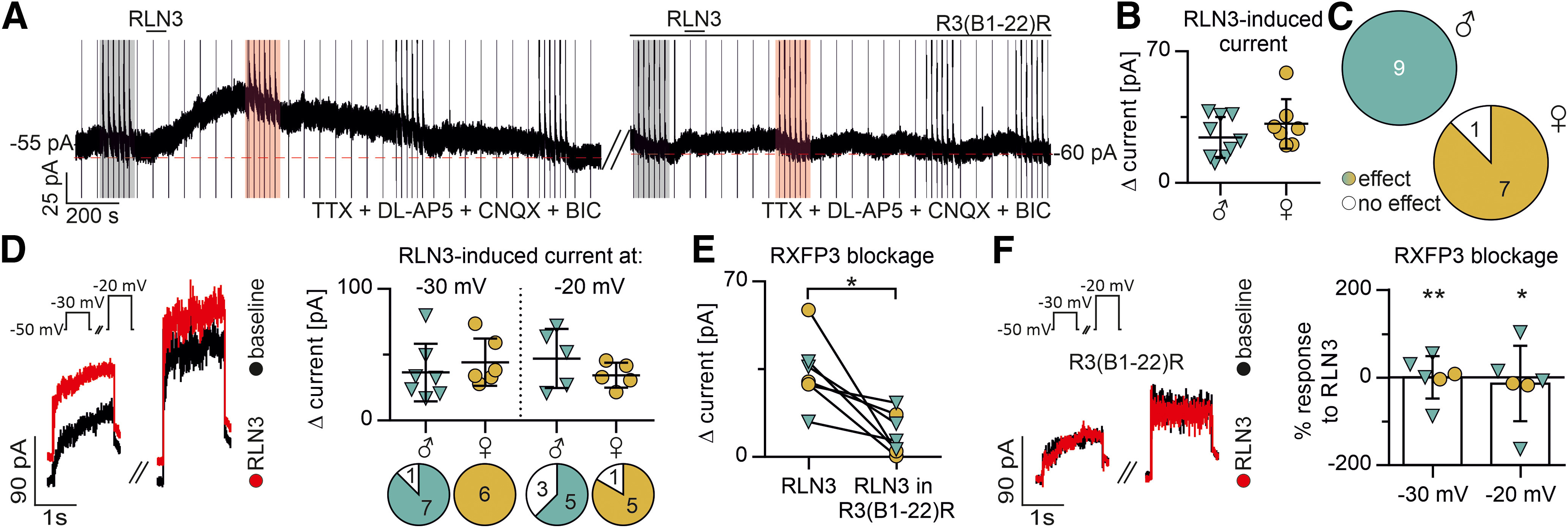
RLN3 induces an RXFP3-dependent outward whole-cell current in a majority of PVN MNCs from male and female rats. ***A***, RLN3-induced outward whole-cell current (voltage-clamp recording, command potential −50 mV) during pharmacological deafferentation (TTX, DL-AP5, CNQX, BIC) and its blockade by a potent RXFP3 antagonist, R3(B1-22)R. Marked areas represent voltage-clamp stimulations to −30 and −20 mV (example shown in ***D***). Black represents baseline. Red represents response to RLN3. ***B***, Amplitude of RLN3-induced outward whole-cell current in MNCs from male and female rats at the command potential of −50 mV. No significant sex difference was observed (unpaired *t* test with Welch's correction, *p* > 0.05). ***C***, Proportion of RLN3-responsive versus nonresponsive MNCs in voltage-clamp recordings. No significant sex difference was observed (Fisher's exact test, *p* > 0.05). ***D***, RLN3-induced whole-cell outward current during voltage stimulation to −30 and −20 mV. Inset, Stimulation protocol. Note the absence of the RLN3-induced effect in the presence of R3(B1-22)R (***F***). Graph represents the amplitude of RLN3-induced current and the proportion of RLN3-responsive male and female MNCs displaying a RLN3-induced change in current at given membrane potentials. Color represents effect. White represents no effect. No significant sex difference was observed in current amplitude (unpaired *t* test with Welch's correction, *p* > 0.05) or in the proportions of MNCs with an RLN3-induced change during voltage stimulations (Fisher's exact test, *p* > 0.05). ***E***, RLN3-induced current amplitude at command potential of −50 mV under control conditions and in the presence of R3(B1-22)R. *p < 0.05, significant influence of treatment with R3(B1-22)R (repeated-measures two-way ANOVA). No significant sex difference or interaction of sex and treatment were observed (*p* > 0.05). ***F***, Blockade of RLN3-induced current during stimulation to −30 and −20 mV in the presence of R3(B1-22)R. Inset, stimulation protocol. Note the absence of RLN3-induced effect. Graph represents the blockade of RLN3-induced current during stimulation expressed as the remaining percentage of the RLN3-induced current amplitude under paired control conditions. *p < 0.05 and **p < 0.01 indicate the significant reduction tested using an unpaired t-test with Welch's correction. All error bars represents SD.

Initially, the responsiveness to RLN3 (100 nm, final bath concentration) was characterized. MNCs (9 MNCs from male and 8 MNCs from female rats) were voltage-clamped at −50 mV and recorded in the presence of the voltage-gated sodium channels blocker, TTX, and the antagonists of ionotropic glutamate and GABA receptors, CNQX, DL-AP5, and bicuculline, to eliminate possible presynaptic and polysynaptic effects. In a vast majority of MNCs (100% of MNCs from male rats and 88% of MNCs from female rats), bath administration of RLN3 enhanced outward whole-cell current recorded at the −50 mV command potential (Fig. 1*A-C*). Additionally, in the presence of RLN3, an augmented outward current (or decreased inward current) was recorded in response to a brief change (1.5 s) in command potential to −30 and −20 mV in a majority of RLN3-responsive MNCs (Fig. 1*D*).

As RLN3 can also bind and activate the relaxin receptor, RXFP1, which is present in the rat PVN, we tested the RXFP3-dependent nature of RLN3 actions. In a subset of neurons (four MNCs from male and three MNCs from female rats), after application of RLN3, the peptide was reapplied in the presence of the potent RXFP3 antagonist, R3(B1-22)R (20 μm) (Haugaard-Kedström et al., 2011). Under these conditions, RLN3 had no effect or the amplitude of the RLN3-induced outward current was substantially reduced; both at the command potential of −50 mV (repeated-measures two-way ANOVA, antagonist treatment: *F*_(1,5)_ = 14,64, df = 1, *p* = 0.0123) and during brief depolarizing pulses to −30 (paired *t* test: *t* = 4.253, df = 5, *p* = 0.0081) and −20 mV (paired *t* test: *t* = 3.912, df = 5, *p* = 0.0113; Fig. 1*A*,*E*,*F*). No sex differences were observed in the proportion of neurons responsive to RLN3 (Fisher's exact test: *p* > 0.05) or the amplitude of RLN3-induced current at any tested command potential (unpaired *t* test with Welch's correction: *p* > 0.05). Similarly, there were no sex differences or interaction in blockage of RLN3-induced current by the RXFP3 antagonist (repeated-measures two-way ANOVA, sex, and interaction of sex and antagonist treatment: *p* > 0.05). Since the influence of RLN3 on MNC electrophysiology was RXFP3-dependent, subsequent experiments were performed using the selective RXFP3 agonist, RXFP3-A2 (600 nm) (Shabanpoor et al., 2012).

**Figure 2.**
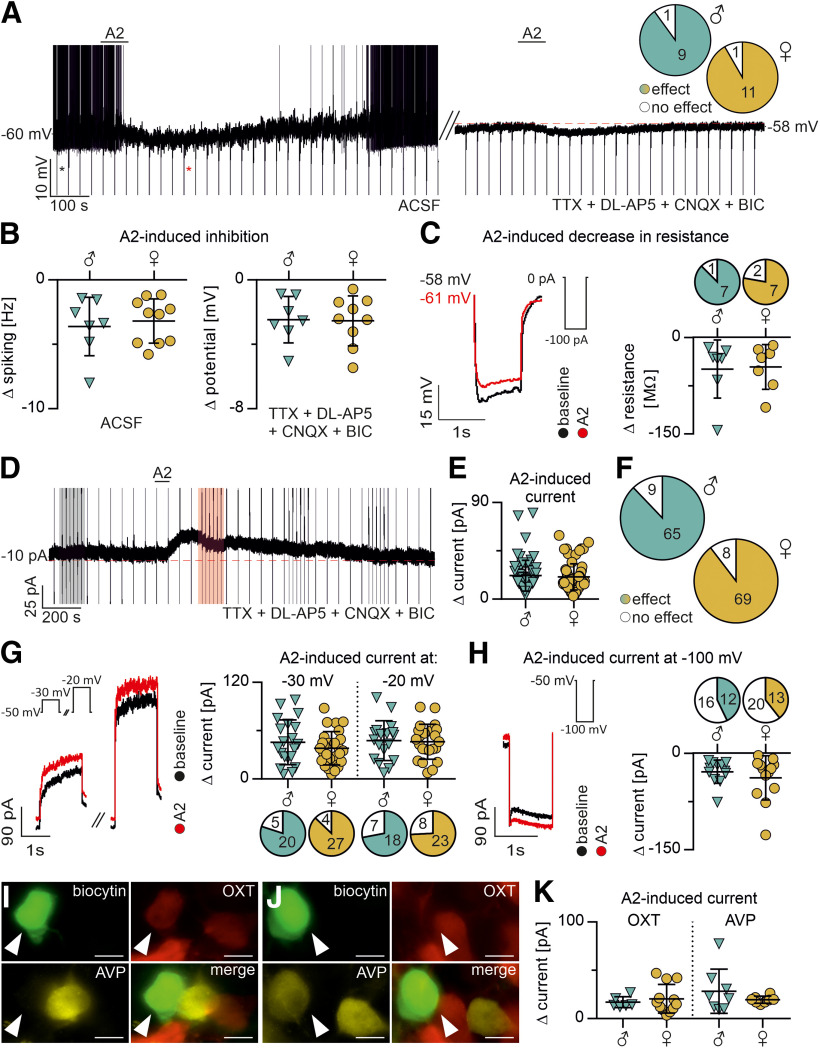
RXFP3 activation with a selective agonist, RXFP3-A2, is inhibitory in the majority of PVN MNCs from male and female rats. ***A***, RXFP3-A2-induced inhibition of spiking and hyperpolarization (current-clamp recording, zero-current mode). Hyperpolarization persists during blockade of spiking and pharmacological deafferentation (in ACSF with TTX, DL-AP5, CNQX, BIC). Inset, The proportion of RXFP3-A2-responsive versus nonresponsive MNCs in current-clamp recordings. No significant sex difference was observed (Fisher's exact test, *p* > 0.05). ***B***, RXFP3-A2-induced reduction of spiking frequency (recorded in normal ACSF), and RXFP3-A2-induced membrane hyperpolarization (recorded in ACSF with TTX, DL-AP5, CNQX, BIC). No significant sex difference in either parameter was observed (unpaired *t* test with Welch's correction, *p* > 0.05). ***C***, RXFP3-A2-induced reduction of voltage responses to hyperpolarizing current pulses indicating a decrease in input resistance. Inset, Stimulation protocol. Traces come from the recording in ***A*** (marked with asterisks). Graph represents the calculated change in input membrane resistance and the proportion of male and female RXFP3-A2-inhibited MCNs displaying an accompanying decrease in input membrane resistance. Color represents effect. White represents no effect. No significant sex difference was observed in the RXFP3-A2-induced change (unpaired *t* test with Welch's correction, *p* > 0.05) or the proportions of MNCs with an RXFP3-A2-induced decrease in input resistance accompanying the RXFP3-A2-induced inhibition (Fisher's exact test, *p* > 0.05). ***D***, RXFP3-A2-induced outward whole-cell current (voltage-clamp recording, command potential −50 mV) during pharmacological deafferentation (TTX, DL-AP5, CNQX, BIC). Marked areas represent voltage-clamp stimulations to −30 and −20 mV (example shown in ***G***). Black represents baseline. Red represents response to RXFP3-A2. ***E***, Amplitude of RXFP3-A2-induced outward whole-cell current in MNCs from male and female rats at command potential of −50 mV. No significant sex difference was observed (unpaired *t* test with Welch's correction, *p* > 0.05). ***F***, Proportion of RXFP3-A2-responsive versus nonresponsive MNCs in voltage-clamp recordings. No significant sex difference was observed (Fisher's exact test, *p* > 0.05). ***G***, RXFP3-A2-induced whole-cell outward current during voltage stimulation to −30 and −20 mV. Inset, Stimulation protocol. Graph represents the amplitude of RXFP3-A2-induced current and proportion of RXFP3-A2-responsive male and female MNCs displaying RXFP3-A2-induced change in whole-cell current at given membrane potentials. Color represents effect. White represents no effect. No significant sex difference was observed in current amplitude (unpaired *t* test with Welch's correction, *p* > 0.05), or in the proportions of MNCs with an RXFP3-A2-induced change during voltage stimulations (Fisher's exact test, *p* > 0.05). ***H***, RXFP3-A2-induced whole-cell inward current during voltage stimulation to −100 mV in a minor subset of MNCs. Inset, stimulation protocol. Graph represents the amplitude of the RXFP3-A2-induced current and proportion of male and female RXFP3-A2-responsive MCNs displaying increased inward whole-cell current at the command potential of −100 mV. Color represents effect. White represents no effect. No significant sex differences were observed in the amplitude of RXFP3-A2-induced current (unpaired *t* test with Welch's correction, *p* > 0.05), or in the proportions of MNCs with RXFP3-A2-induced change during voltage stimulations (Fisher's exact test, *p* > 0.05). ***I***, ***J***, Series of fluorescent projection images of biocytin-filled OXT-ir (in ***I***) or AVP-ir (in ***J***) MNCs (white arrowhead). Scale bar, 10 µm. ***K***, Amplitude of RXFP3-A2-induced outward whole-cell current (command potential −50 mV) in OXT and AVP MNCs from male and female rats. No significant effects of peptide content, sex, and interaction of peptide content and sex were observed in the amplitude of RXFP3-A2-induced outward whole-cell current (two-way ANOVA, *p* > 0.05). All error bars represents SD.

The influence of selective RXFP3 activation on spontaneous PVN MNC activity was investigated in normal ACSF in a zero current-clamp mode (10 MNCs from male and 12 MNCs from female rats). The majority of MNCs were inhibited by RXFP3-A2 (90% of male and 92% of female MNCs; Fig. 2*A*); bath application of the peptide resulted in a reduction or cessation of firing and/or membrane hyperpolarization (Fig. 2*A*,*B*). The RXFP3-induced inhibition of neuronal activity was accompanied by a decrease in membrane resistance in a majority of responsive neurons, pointing to an increase in the ionic conductance underlying the observed hyperpolarization (in 88% of male and 78% of female MNCs; Fig. 2*C*). To verify the postsynaptic character of RXFP3-induced inhibition, a subset of RXFP3-A2-responsive MNCs (7 MNCs from male and 9 MNCs from female rats) was reexposed to the drug in the presence of TTX, CNQX, DL-AP5, and bicuculline. All tested MNCs remained responsive to RXFP3-A2 under these conditions (Fig. 2*A*,*B*). Again, no sex differences were observed in the proportions of RXFP3-A2-responsive MNCs (Fisher's exact test: *p* > 0.05), agonist-evoked change in spiking frequency, membrane potential, or input resistance (unpaired *t* test with Welch's correction: *p* > 0.05).

Next, the influence of selective RXFP3 activation on MNC whole-cell current was tested. MNCs (74 from male and 77 from female rats) were voltage-clamped at a command potential of −50 mV and recorded in ACSF containing TTX, CNQX, DL-AP5, and bicuculline. Under these conditions, RXFP3-A2 induced an outward whole-cell current in a majority of tested MNCs (in 88% of male and 90% of female MNCs; Fig. 2*D-F*). Moreover, current responses to brief (1.5 s) changes of command potential to −30 and −20 mV were analyzed in a subset of neurons (25 MNCs from male and 31 MNCs from female rats). In a majority of RXFP3-A2-responsive MNCs, an increased outward current (or decreased inward current) was observed in a whole-cell current recorded at these potentials during the agonist action (Fig. 2*G*). Additionally, we analyzed current responses to brief (1 s) hyperpolarizing steps to a command potential of −100 mV in a subset of neurons (28 MNCs from male and 33 MNCs from female rats). In a majority of RXFP3-A2-sensitive MNCs (57% males and 61% females), no change was observed in whole-cell current recorded at −100 mV during the agonist action (Fig. 2*H*). As in our recording conditions, the calculated reversal potential for K^+^ equals −102 mV (similar to the command potential during hyperpolarizing steps), a lack of RXFP3-A2-induced current at this potential, with a simultaneous robust effect present at −50 mV, points to the possible involvement of a K^+^ conductance in the action of RXFP3-A2. In contrast, in the remaining, smaller subset of RXFP3-A2-sensitive MNCs (43% in males and 39% in females), the outward current induced by RXFP3 activation at −50 mV was accompanied by an increase in the inward current recorded at −100 mV, pointing to activation of an additional excitatory conductance in some MNCs (Fig. 2*H*). Again, no sex differences were observed either in the proportion of neurons responsive to RXFP3-A2 (Fisher's exact test: *p* > 0.05) or the amplitude of RXFP3-A2-induced current at any command potential (unpaired *t* test with Welch's correction: *p* > 0.05).

Postrecording immunohistochemical identification of MNCs revealed that both OXT-ir and AVP-ir MNCs were sensitive to RXFP3-A2 (Fig. 2*I*,*J*). No differences were observed among male and female OXT-ir and AVP-ir MNCs in the amplitude of the RXFP3-A2-induced current (repeated-measures two-way ANOVA, peptide content, sex, and interaction of peptide content and sex: *p* > 0.05; Fig. 2*K*). Therefore, data from all MNCs were pooled.

### RXFP3-mediated inhibition is caused by a potassium conductance resembling an M-current

After observing the effect of the RXFP3-A2 peptide and its termination and washout, RXFP3-A2 was reapplied under several experimental conditions to investigate the ionic mechanism(s) underlying the RXFP3-induced inhibition of PVN MNCs. As no statistically significant sex differences or interactions of sex and pharmacological treatment were observed in any of these experiments (repeated-measures two-way ANOVA, sex and interaction of sex and pharmacological treatment: *p* > 0.05), data obtained from male and female rats were pooled.

**Figure 3.**
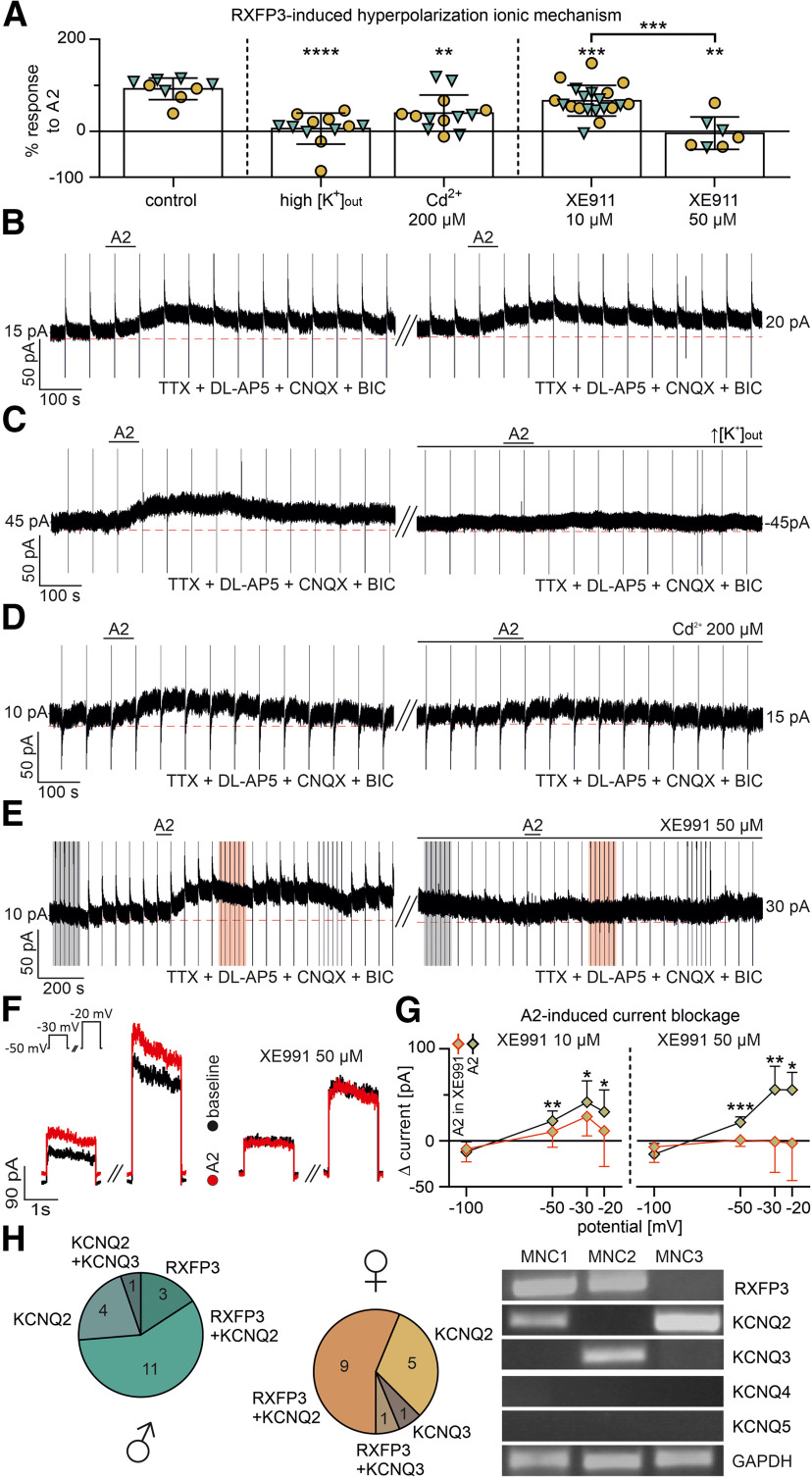
RXFP3 activation induces M-like potassium current. ***A***, RXFP3-A2-induced outward current at a command potential of −50 mV under several different conditions expressed as a remaining percentage of the RXFP3-A2-induced current amplitude under paired control conditions. Triangle represents male. Circle represents female. **p < 0.01; ***p < 0.001; ****p < 0.0001; significant influence of treatment with the compound (repeated-measures two-way ANOVA). No significant sex differences or interaction of sex and treatment were observed (*p* > 0.05). Note the significant dose-dependent effect of XE991. ***p <0.001, significant influence of the XE991 concentrations (two-way ANOVA). No significant sex differences or interaction of sex and XE991 concentration were observed (*p* > 0.05). ***B-E***, RXFP3-A2-induced outward whole-cell current (command potential −50 mV) during pharmacological deafferentation (TTX, DL-AP5, CNQX, BIC) and reexposure to RXFP3-A2 under several conditions: control (repeated RXFP3 application with no additional compound, in ***B***) increased extracellular [K^+^] in the ACSF (in ***C***), presence of Cd^2+^ (200 μm, in ***D***), and presence of XE991 (50 μm, in ***E***). ***E***, Marked areas represent voltage-clamp stimulations to −30 and −20 mV (example shown in ***F***). Black represents baseline. Red represents response to RXFP3-A2. ***F***, RXFP3-A2-induced whole-cell outward current during voltage stimulation to −30 and −20 mV. Inset, Stimulation protocol. Note the absence of the RXFP3-A2-induced effect in the presence of XE991 (50 μm). ***G***, The I--V relationship of RXFP3-A2-induced outward whole-cell current under control conditions and in the presence of XE991 (10 and 50 μm, respectively). Note the dose-dependent blockade of RXFP3-A2-induced current by XE991. *p < 0.05; **p < 0.01; ***p < 0.001; significant influence of treatment with XE991 (repeated-measures two-way ANOVA). No significant sex differences or interaction of sex and treatment were observed (*p* > 0.05). ***H***, Proportions of male and female MNCs expressing and coexpressing RXFP3 and KCNQ subunit mRNA detected by sc-RT-PCR. Only MNCs with detectable GAPDH mRNA were analyzed. KCNQ4 and KCNQ5 mRNA was not detected in our PVN MNCs preparations. No significant sex difference in these proportions was observed (χ^2^ test, *p* > 0.05). Image represents exemplary sc-RT-PCR data from three female MNCs. All error bars represents SD.

Reexposure to RXFP3-A2 under the same conditions as the first application (ACSF containing TTX and blockers of synaptic transmission) induced an outward current of a comparable magnitude, consistent with a lack of RXFP3 desensitization or internalization following initial or repeated stimulations (9 MNCs, repeated-measures two-way ANOVA, repeated agonist treatment: *p* > 0.05; Fig. 3*A*,*B*).

To verify the hypothesis that RXFP3 activation leads to increased potassium conductance, a modified ACSF containing an elevated K^+^ concentration (final concentration: 21.6 mm, with a simultaneously reduced NaCl concentration) was used in this subset of experiments. Under these conditions, EK equaled the command potential (−50 mV), and the RXFP3-A2 effect was almost completely abolished (12 MNCs, 94% reduction in RXFP3-A2-induced current amplitude compared with the first peptide application, repeated-measures two-way ANOVA, high K^+^ treatment: *F*_(1,10)_ = 32.89, df = 1, *p* = 0.0002; Fig. 3*A*,*C*).

The RXFP3-A2-induced current was also reduced in the presence of cadmium ions (Cd^2+^), a blocker of Ca^2+^ conductances (Hille, 2001), confirming our previous observation in Wistar male rats (Kania et al., 2017). Addition of CdCl_2_ (200 μm) to the bathing solution reduced the amplitude of RXFP3-A2-induced current by 60% compared with the effect produced by the first application (13 MNCs, repeated-measures two-way ANOVA, Cd^2+^ treatment: *F*_(1,11)_ = 11.39, df = 1, *p* = 0.0062; Fig. 3*A*,*D*). Therefore, these data suggest that the RXFP3-A2-induced potassium current might be Ca^2+^-dependent.

Next, as the presence of a Ca^2+^-dependent M-like conductance was reported in MNCs (W. Zhang et al., 2009), we tested whether the RXFP3-A2-induced current could be blocked by the M-current inhibitor XE991 (Jentsch, 2000). Indeed, XE991 dose-dependently blocked the current activated by the RXFP3 activation: 10 μm XE991 reduced its amplitude by 34% (21 MNCs, repeated-measures two-way ANOVA, 10 μm XE991 treatment: *F*_(1,18)_ = 17.30, df = 1, *p* = 0.0006), whereas 50 μm XE991 reduced it by 104% compared with the first peptide application (8 MNCs, repeated-measures two-way ANOVA, 50 μm XE991 treatment: *F*_(1,6)_ = 24.05, df = 1, *p* = 0.0027; XE991: 10 μm vs 50 μm, two-way ANOVA, XE991 concentration: *F*_(1,23)_ = 20.73, df = 1, *p* = 0.0001; Fig. 3*A*,*E*,*F*). In one MNC, the blockade of the RXFP3-A2-induced outward current with XE991 (10 μm) revealed a strong RXFP3-A2-induced inward current. This cell was excluded from the analysis.

In a group of MNCs recorded at a command potential of −50 mV, with brief voltage stimulations to −100 mV (1 s), as well as −30 and −20 mV (1.5 s) throughout recording, an I--V relationship of RXFP3-A2-induced current and its blockade by XE991 was analyzed (14 MNCs treated with 10 μm XE991 and 6 MNCs treated with 50 μm XE991). Both concentrationsof XE991 dose-dependently blocked the RXFP3-A2 induced current in a potential-dependent manner (RXFP3-A2 effect blockade with 10 μm XE991, 14 MNCs, repeated-measures two-way ANOVA, 10 μm XE991 treatment for: −100 mV, *p* > 0.05; −50 mV, *F*_(1,12)_ = 10.68, df = 1, *p* = 0.0067; −30 mV, *F*_(1,12)_ = 6.259, df = 1, *p* = 0.0278; −20 mV, *F*_(1,12)_ = 6.259, df = 1, *p* = 0.0278; RXFP3-A2 effect blockade with 50 μm XE991, 6 MNCs, repeated-measures two-way ANOVA, 50 μm XE991 treatment for: −100 mV, *p* > 0.05; −50 mV, *F*_(1,4)_ = 76.29, df = 1, *p* = 0.0009; −30 mV, *F*_(1,4)_ = 37.18, df = 1, *p* = 0.0037; −20 mV, *F*_(1,4)_ = 16.71, df = 1, *p* = 0.0150; Fig. 3*E-G*).

The neural M-current is mainly mediated by ionic channels composed by four members of the KCNQ transmembrane subunit family (KCNQ2-5), with different homodimer andheterodimer combinations forming channels of varying electrical and pharmacological properties (for review, see Jentsch, 2000; Robbins, 2001; Brown and Passmore, 2009). Expression of KCNQ subunits has been observed in PVN (Zhou et al., 2017) and MNCs of SON (W. Zhang et al., 2009); therefore, using sc-RT-PCR, we examined possible coexpression of RXFP3 and KCNQ channel subunits in the PVN MNCs to determine possible molecular substrates for the modulation of the M-current by the RLN3/RXFP3 signaling. After whole-cell recordings, the cytoplasm of single MNCs was aspirated into the 1-2 MΩ resistance patch micropipettes and used for sc-RT-PCR. A combination of specific primers was used to detect several mRNA species: RXFP3, KCNQ2-5, and GAPDH (only samples verified to express GAPDH mRNA were analyzed). A total number of 40 MNCs (21 from males and 19 from females) were included in this analysis. We observed the frequent presence of RXFP3 mRNA (in 67% male and 53% female MNCs) and KCNQ2 mRNA (in 76% male and 74% female MNCs), and individual MNCs expressing KCNQ3 mRNA (1 neuron from male and 2 neurons from female MNCs), whereas KCNQ4 and KCNQ5 mRNA were not detected (Fig. 3*H*). Notably, the majority of KCNQ2 mRNA-expressing MNCs coexpressed RXFP3 mRNA (69% male and 64% female MNCs). No sex difference in the proportions of MNCs expressing specific mRNA species was observed (χ^2^ test, *p* > 0.05).

### Synaptic input to PVN MNCs is unaffected by RXFP3 activation

In a separate series of whole-cell experiments, we investigated the possible effect of RLN3 (100 nm) and RXFP3-A2 (600 nm) on spontaneous and miniature synaptic currents in MNCs (both excitatory and inhibitory). Neurons were voltage-clamped at −50 mV, and synaptic currents were recorded in normal or TTX-containing ACSF. Separate subsets of neurons were tested with either RLN3 (20 MNCs from male and 19 MNCs from female rats) or RXFP3-A2 (20 MNCs from male and 17 MNCs from female rats). Neither peptide significantly influenced any of the following parameters of spontaneous/miniature synaptic activity: frequency, amplitude, rise time, or decay time constant of both IPSCs and EPSCs (repeated-measures two-way ANOVA, RLN3 or RXFP3-A2 treatment, sex, and interaction of RLN3 or RXFP2-A2 treatment and sex: *p* > 0.05; Table 2).

**Table 2.** Synaptic input to PVN MNCs is unaffected by RLN3 and a selective RXFP3 agonist*^a^*

	Inhibitory				Excitatory			
	Male (*N* = 11)		Female (*N* = 9)		Male (*N* = 11)		Female (*N* = 9)	
	Baseline	RLN3	Baseline	RLN3	Baseline	RLN3	Baseline	RLN3
**RLN3**								
Spontaneous synaptic activity								
Frequency (Hz)	8.9 ± 5.4	9.2 ± 5.7	7.8 ± 5.9	7.9 ± 5.7	1.3 ± 1.0	1.2 ± 0.6	0.8 ± 0.3	0.9 ± 0.4
Mean amplitude (pA)	33.6 ± 9.6	32.7 ± 9.9	31.0 ± 5.4	30.2 ± 4.6	18.9 ± 2.7	18.9 ± 3.4	21.6 ± 3.5	21.3 ± 3.5
Mean rise time (ms)	2.8 ± 0.4	2.8 ± 0.3	2.7 ± 0.3	2.7 ± 0.2	2.4 ± 0.2	2.3 ± 0.1	2.4 ± 0.2	2.4 ± 0.2
Decay time constant (ms)	5.5 ± 1.3	5.6 ± 1.3	5.0 ± 1.2	4.9 ± 1.0	2.3 ± 0.8	2.3 ± 0.6	2.1 ± 0.5	2.0 ± 0.5
	Male (*N* = 9)		Female (*N* = 10)		Male (*N* = 9)		Female (*N* = 10)	
	Baseline	RLN3	Baseline	RLN3	Baseline	RLN3	Baseline	RLN3

Miniature synaptic activity								
Frequency (Hz)	10.1 ± 6.0	9.5 ± 5.3	6.4 ± 4.4	6.4 ± 4.5	1.5 ± 1.6	1.1 ± 0.6	0.9 ± 0.4	0.9 ± 0.4
Mean amplitude (pA)	25.8 ± 3.8	26.1 ± 4.0	26.1 ± 5.6	25.7 ± 5.3	19.6 ± 5.3	18.9 ± 3.6	16.3 ± 3.4	16.3 ± 3.2
Mean rise time (ms)	2.6 ± 0.2	2.6 ± 0.2	2.6 ± 0.2	2.6 ± 0.2	2.3 ± 0.1	2.3 ± 0.1	2.3 ± 0.2	2.4 ± 0.1
Decay time constant (ms)	4.7 ± 0.8	4.7 + 0.7	4.9 ± 0.9	5.1 ± 0.8	2.1 ± 0.9	1.8 ± 0.7	2.0 ± 0.6	2.0 ± 0.5
	Male (*N* = 10)		Female (*N* = 9)		Male (*N* = 10)		Female (*N* = 9)	
	Baseline	A2	Baseline	A2	Baseline	A2	Baseline	A2

**RXFP3-A2**								
Spontaneous synaptic activity								
Frequency (Hz)	8.6 ± 7.3	8.4 ± 7.1	11.3 ± 6.7	11.0 ± 6.5	1.0 ± 0.8	1.0 ± 0.6	1.1 ± 0.6	1.1 ± 0.5
Mean amplitude (pA)	32.0 ± 5.5	31.8 ± 6.3	34.1 ± 7.4	33.0 ± 7.2	19.5 ± 4.8	20.9 ± 6.3	19.7 ± 2.3	20.0 ± 3.0
Mean rise time (ms)	2.8 ± 0.3	2.7 ± 0.2	2.8 ± 0.2	2.8 ± 0.2	2.4 ± 0.2	2.4 ± 0.2	2.3 ± 0.1	2.3 ± 0.1
Decay time constant (ms)	5.9 ± 1.1	5.7 ± 1.1	5.8 ± 1.3	5.9 ± 1.2	3.0 ± 1.6	2.7 ± 1.2	2.1 ± 0.5	2.1 ± 0.4
	Male (*N* = 10)		Female (*N* = 8)		Male (*N* = 10)		Female (*N* = 8)	
	Baseline	A2	Baseline	A2	Baseline	A2	Baseline	A2

Miniature synaptic activity								
Frequency (Hz)	6.8 ± 3.2	6.7 ± 3.0	7.8 ± 3.7	7.3 ± 3.7	1.0 ± 0.5	1.1 ± 0.5	0.7 ± 0.4	0.7 ± 0.4
Mean amplitude (pA)	28.7 ± 5.7	29.2 ± 5.6	32.5 ± 3.2	32.8 ± 3.5	19.6 ± 4.1	19.8 ± 3.2	19.4 ± 1.1	19.3 ± 2.5
Mean rise time (ms)	3.1 ± 0.4	3.0 ± 0.3	3.1 ± 0.8	3.0 ± 0.3	2.8 ± 0.3	2.9 ± 0.2	2.8 ± 0.2	2.8 ± 0.2
Decay time constant (ms)	4.7 ± 1.1	4.7 ± 0.9	4.0 ± 0.5	4.2 ± 0.4	2.2 ± 1.1	2.1 ± 1.0	2.0 ± 0.7	1.9 ± 0.4

*^a^*Spontaneous and miniature postsynaptic currents recorded from male and female MNCs under two experimental conditions: baseline and after application of RLN3 or RXFP3-A2. No significant effect of treatment, sex, or interaction of treatment and sex were observed in all of the cases analyzed (repeated-measures two-way ANOVA, *p* > 0.05).

### RLN3 neurons innervate the vicinity of the PVN

**Figure 4.**
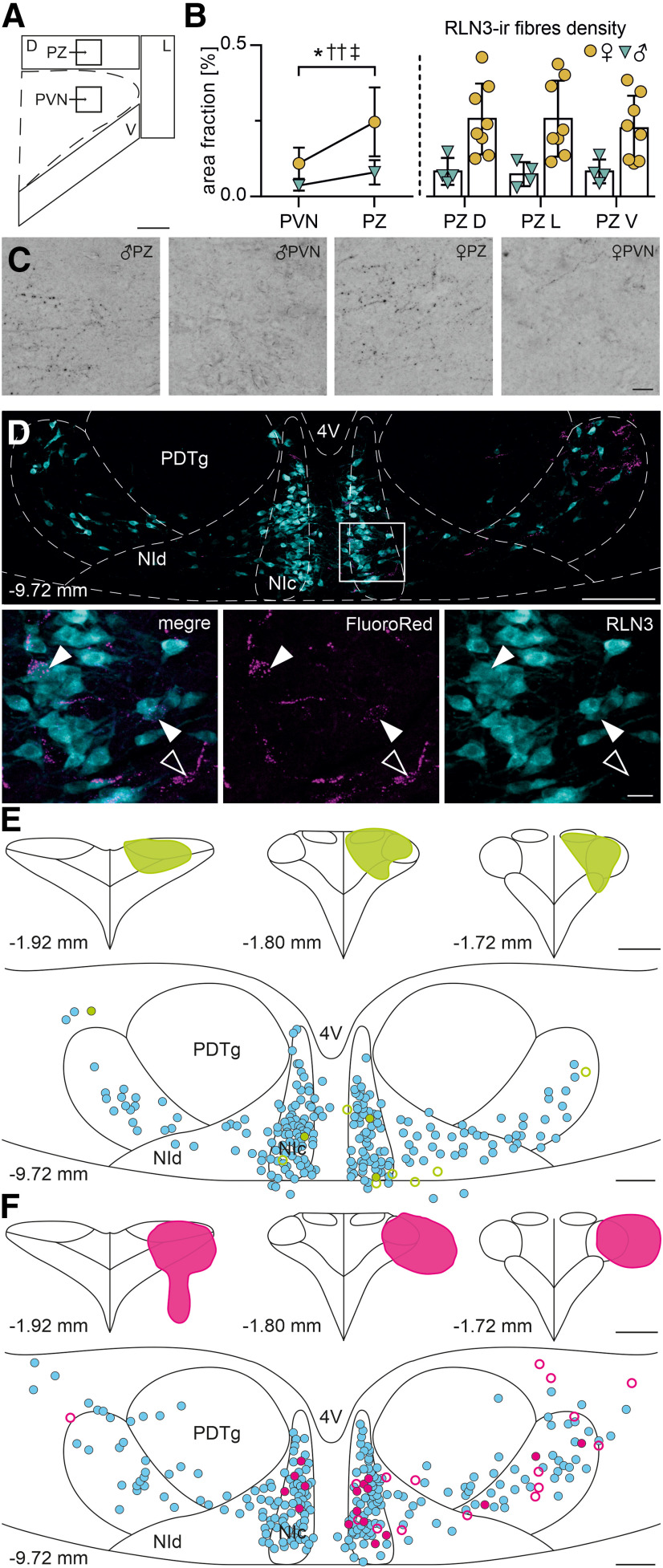
NI RLN3 neurons innervate the PVN and its adjacent areas, with a higher fiber density in female than in male rats. ***A***, Schematic representation of areas analyzed for DAB-stained RLN3 fibers in the PVN and adjacent perinuclear zone (PZ). Scale bar, 200 µm. ***B***, Area fraction of RLN3-ir fibers in the analyzed areas. Between-subjects statistical testing with repeated-measures two-way ANOVA revealed the significant effect of the area of interest (PVN vs PZ, *p < 0.05), sex (males vs females, ††*p* <0.01), as well as interaction (‡*p* < 0.05), pointing to a higher density of RLN3-ir fibers in the peri-PVN zone than in the PVN and in females than in males, with a more profound sex difference observed in the peri-PVN zone. ***C***, Light microscopic projection images of DAB-stained RLN3-ir fibers in the PVN and PZ area of male (left) and female (right) rats. Images are magnifications of the areas marked with black squares in ***A***. Note the difference between the PVN and the peri-PVN zone. Scale bar, 20 µm. ***D***, Confocal projection image illustrating RLN3-ir neurons (cyan) and neurons filled with retrograde tracer (FluoroRed, magenta) in the NI. Magnification of the area indicated by a white rectangle represents RLN3-ir neurons, RLN3-ir/FluoroRed-positive neurons (white arrowheads), and RLN3-negative/FluoroRed-positive neuron (empty arrowhead). Distance from bregma indicated. Scale bars: top, 200 µm; bottom, 20 µm. ***E***, ***F***, Exemplary injection sites of fluorescent retrograde tracers (FluoroGreen in ***E*** and FluoroRed in ***F***) and reconstructions of single NI sections showing the distribution of RLN3-ir neurons (cyan circles), RLN3-ir/retrograde tracer-positive (green circles in ***E*** and red circles in ***F***), and RLN3-negative/retrograde tracer-positive (open green circles in ***E*** and open red circles in ***F***). The injection in ***E*** is restricted to the PVN, whereas the injection in ***F*** extends beyond the PVN borders, resulting in different number of RLN3-ir/retrograde tracer-positive between these two injections. Distance from bregma indicated. Scale bars: PVN reconstructions, 400 µm; NI reconstructions, 200 µm. 4V, Fourth ventricle; D, dorsal perinuclear zone of the PVN; L, lateral perinuclear zone of the PVN; NIc, NI pars compacta; NId, NI pars dissipata; PDTg, posterodorsal tegmental nucleus; PZ, perinuclear zone of the PVN; V, ventral perinuclear zone of the PVN. All error bars represents SD.

Our previous studies in male Wistar rats revealed that dense RLN3-ir fibers surround the PVN, and only rare fibers enter the main soma-rich nucleus area (Kania et al., 2017). Here, to further characterize the anatomy of the RLN3 input to the PVN in male and female Sprague Dawley rats, we performed immunohistochemical and neural tract-tracing studies. Coronal hypothalamic sections throughout the PVN from males and females were immunostained to visualize RLN3-ir fibers. We observed an abundance of RLN3-ir fibers at the PVN border proximity and scarce RLN3 innervation of the nucleus itself. Quantitative analysis of fiber densities within the PVN and the peri-PVN zone revealed statistically significant differences (Fig. 4*A-C*). Moreover, ANOVA revealed sex differences and interaction of sex and analyzed area (PVN vs peri-PVN zone) with females displaying a higher fiber density than males and the difference being more profound in the perinuclear zone (repeated-measures two-way ANOVA, PVN vs peri-PVN zone: *F*_(1,10)_ = 20.55, df = 1, *p* = 0.0011; sex: *F*_(1,10)_ = 8.190, df = 1, *p* = 0.0169; interaction of PVN vs peri-PVN zone and sex: *F*_(1,10)_ = 5.429, df = 1, *p* = 0.0421).

In a series of double neural tract-tracing experiments, FluoroGreen and FluoroRed retrograde tracers were injected bilaterally into the PVN area of male and female Sprague Dawley rats. Coronal sections from all rat brain areas where RLN3-synthesizing neurons are located (PAG, PnR, dSN, and NI) were immunostained for RLN3 and examined for colocalization of tracer and peptide. Sparse retrogradely filled RLN3-ir neurons were observed in the NI, whereas only individual retrogradely labeled RLN3-ir neurons were present in the PAG, PnR, and dSN (Table 3; Fig. 4*D*). In line with the low RLN3 fiber density in the PVN and high density in the perinuclear zone, the number of tracer-positive RLN3-ir neurons depended on the size and precise localization of the tracer injection site. Injections restricted to the PVN area revealed a more scarce RLN3 innervation (mainly NI originating), than tracer injections extending beyond the PVN soma boundary, which resulted in considerably higher number of retrogradely filled, RLN3-positive neurons (Table 3; Fig. 4*E*,*F*). Additionally, the mean number of tracer-positive RLN3-ir neurons was highest when the injections beyond the PVN border were localized in the mid-region of the PVN anterior-posterior axis, and lower after perinuclear zone injections restricted to the posterior part of the nucleus (Table 3). Notably, the number of retrogradely labeled neurons was substantially higher ipsilateral to the injection site. This lateralization was most profound in the NI (Table 3; Fig. 4*E*,*F*).

**Table 3.** RLN3 neurons innervate PVN and its vicinity*^a^*

Tracer injection site		Retrogradely traced RLN3-ir neurons							
		PAG		dSN		PnR		NI	
		Contralateral	Ipsilateral	Contralateral	Ipsilateral	Contralateral	Ipsilateral	Contralateral	Ipsilateral
PVN	Males (*N* = 3)	3 ± 3	3 ± 3	2 ± 3	0	0	0	6 ± 8	14 ± 15
	Females (*N* = 1)	3	9	3	0	0	0	6	12
PERI-PVN	Males (*N* = 3)	3 ± 3	7 ± 5	2 ± 2	5 ± 5	2 ± 2	1 ± 2	40 ± 44	98 ± 76
	Females (*N* = 7)	6 ± 4	9 ± 8	1 ± 2	4 ± 3	2 ± 2	5 ± 4	30 ± 16	76 ± 46
PERI-PVN posterior	Females (*N* = 3)	1 ± 2	4 ± 3	1 ± 2	1 ± 2	1 ± 2	2 ± 3	9 ± 9	25 ± 18

*^a^*The numbers of rln3-ir/retrograde tracer-positive neurons in all known brain areas that synthesize RLN3 in male and female rats, with regard to the injection site. Note the substantially higher number of RLN3-ir/retrograde tracer-positive neurons ipsilaterally to the injection sites, as well as in cases where tracer injections extended beyond the PVN.

No qualitative sex differences were observed in the source of the RLN3 innervation of the PVN and its vicinity, with PVN-innervating RLN3 neurons present in all examined areas in male and female rats.

### RXFP3 activation in the PVN is necessary for the occurrence of BE behavior

**Figure 5.**
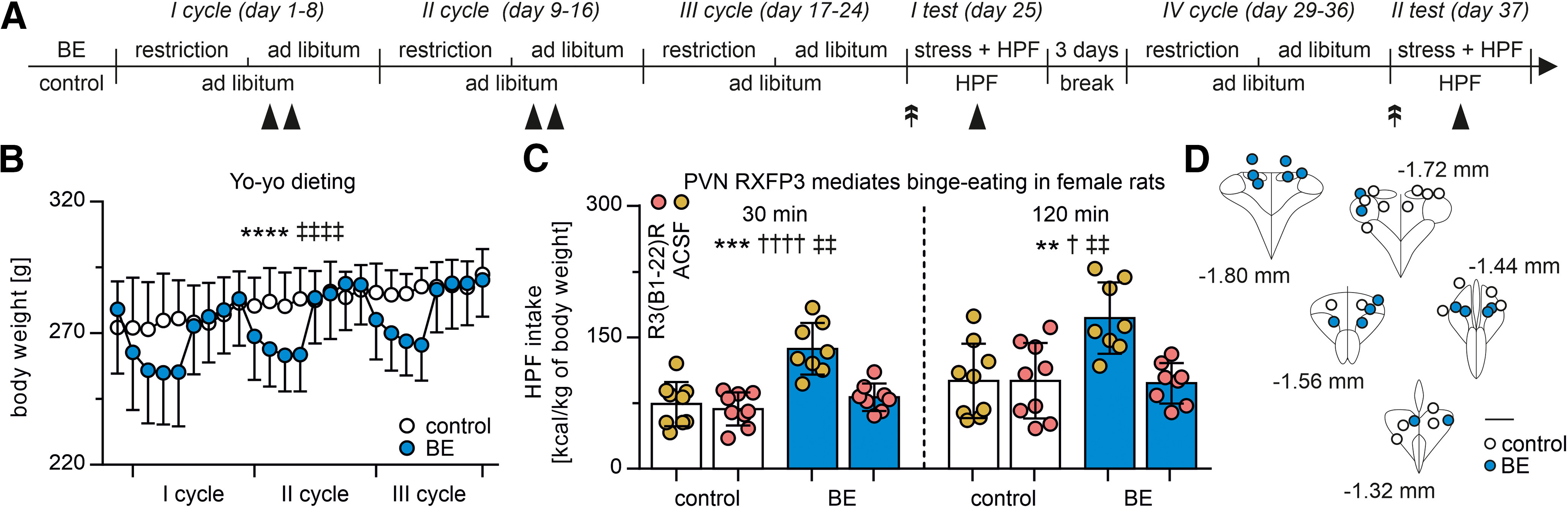
Injection of an RXFP3 antagonist into the PVN reduced BE in female rats. ***A***, Timeline of the BE induction protocol. Rats in the BE group were subjected to three cycles of food restriction (66% of regular chow intake) and refeeding (*ad libitum* chow), whereas the control group had *ad libitum* access to chow. Black arrowheads indicate access to the HPF for 2 h in both groups. Double arrows indicate bilateral intra-PVN injections of vehicle (0.5 µl) or R3(B1-22)R (0.1 µg/0.5 µl) made on the test day. In the BE group, injections were followed by frustration stress procedure for 15 min, and both groups had 2 h access to the HPF afterward. After an additional cycle of restriction and refeeding, the test was repeated with reversed injection conditions, so that at the end of the study, each rat received both vehicle and R3(B1-22)R in separate tests. ***B***, Body weights of rats subjected (BE) or not (control) to cycles of intermittent food restriction followed by refeeding. Between-subjects statistical testing with a repeated-measures two-way ANOVA revealed the significant effect of time (****p < 0.0001) and interaction of time and group (‡‡‡‡*p* < 0.0001), with no differences observed between groups, pointing to time-dependent changes with no permanent differences in body weight between feeding conditions. ***C***, HPF intake at 30 and 120 min during the BE tests and the effect of intra-PVN injection of the RXFP3 antagonist, R3(B1-22)R. Between-subjects statistical testing with a two-way ANOVA revealed a significant effect of the treatment (vehicle vs R3(B1-22)R, **p <0.01, ***p < 0.001), experimental group (control vs BE, †*p* < 0.05, ††††*p* < 0.0001), and interaction (‡‡*p* < 0.01), pointing to a higher HPF intake in BE than control rats and the effectiveness of RXFP3 antagonism to reduce the HPF intake only in the BE group. ***D***, Reconstruction of the PVN area on coronal brain sections representing the intra-PVN injections sites in rats included in the experiment. Distance from bregma indicated. Scale bar, 400 µm. All error bars represents SD.

RLN3/RXFP3 signaling has been linked to binge-like eating (Lenglos et al., 2013; Calvez et al., 2016b); so to further assess the involvement of PVN RXFP3 in this phenomenon, we used an established rat model of BE (Cifani et al., 2009; Piccoli et al., 2012; Micioni Di Bonaventura et al., 2017) combined with intra-PVN injections of the RXFP3 antagonist, R3(B1-22)R. This model utilizes yo-yo dieting and exposure to frustration stress in female Sprague Dawley rats (body weight of rats subjected to cycles of food restriction fluctuates but does not differ on test days, repeated-measures two-way ANOVA, restricted vs nonrestricted group: *p* > 0.05; time: *F*_(24,432)_ = 34.81, df = 28, *p* < 0.0001; interaction of restricted vs nonrestricted group and time: *F*_(24,432)_ = 13.03, df = 28, *p* < 0.0001; Fig. 5*A*,*B*) to produce binging on HPF (Fig. 5C). We observed that bilateral intra-PVN injections of R3(B1-22)R (0.1 µg in 0.5 µl of ACSF into each side of the PVN) potently reduced the amount of HPF consumed during a 2 h access period in BE rats, with no effect on consumption by control rats (not exposed to cycles of food restriction and frustration stress; 30 min from access to the HPF: two-way ANOVA, antagonist treatment: *F*_(1,30)_ = 14.91, df = 1, *p* = 0.0006; BE vs control group: *F*_(1,30)_ = 23.45, df = 1, *p* < 0.0001, interaction of antagonist treatment and BE vs control group: *F*_(1,30)_ = 9.919, df = 1, *p* = 0.0037; 120 min from access to the HPF: two-way ANOVA, antagonist treatment: *F*_(1,30)_ = 8.187, df = 1, *p* = 0.0076; BE vs control group: *F*_(1,30)_ = 7.010, df = 1, *p* < 0.0128, interaction of antagonist treatment and BE vs control group: *F*_(1,30)_ = 8.178, df = 1, *p* = 0.0076). Injection sites were histologically verified after completion of the experiment (Fig. 5*D*). Only data from rats with correctly targeted injections were analyzed.

## Discussion

In these studies, we discovered that the inhibitory effect of RXFP3 activation on the activity of PVN MNCs in rats of both sexes is mediated by an M-like K^+^ current. Moreover, we showed that RLN3 fibers are far less abundant in the PVN soma-rich area than in the adjacent perinuclear zone region, suggesting RLN3 actions via non-somatic sites and/or via volume transmission. Notably, higher intra- and peri-PVN RLN3-ir fiber densities were observed in females, a possible anatomic substrate for sex differences in susceptibility to BED. Finally, we demonstrated that RXFP3 blockade in the PVN abolishes BE of HPF in female rats.

Electrophysiological data and pharmacological evidence from the current study indicate that the RXFP3-induced inhibition of the PVN MNCs is mediated by activation of an M-like current. The M-current is a voltage-dependent, slowly activating and noninactivating K^+^ conductance, contributing to the resting membrane potential and to action potential threshold; and is involved in the regulation of membrane excitability (for review, see Brown, 1988; Brown and Passmore, 2009; Greene and Hoshi, 2017). In agreement with our previous study in male Wistar rats (Kania et al., 2017), we confirmed that, in Sprague Dawley rats, the RXFP3-induced outward current is attenuated by external Cd^2+^, a potent blocker of Ca^2+^ channels and Ca^2+^-dependent conductance (Hille, 2001).

The influence of Ca^2+^ on the M-current is complex and depends on the Ca^2+^ concentration and the signaling cascade involved, with calmodulin-KCNQ interactions and Ca^2+^-dependent suppression of M-currents being extensively studied (Marrion, 1997; Delmas and Brown, 2005). In contrast, preventing Ca^2+^ influx with dihydropyridine-type, L-type Ca^2+^ channel blockers suppressed the M-current in hippocampal neurons, whereby 10 μm nimodipine reduced the M-current amplitude by 60% (Wu et al., 2008). Notably, 10 μm nifedipine reduced the amplitude of M-currents in SON MNCs (W. Zhang et al., 2009), and these authors reported that Cd^2+^ significantly reduced the amplitude of an osmosensitive M-like current in MNCs. In the present study, we observed an increase in inward whole-cell current recorded at the hyperpolarized membrane potential (−100 mV) after RXFP3 activation in a subpopulation of PVN MNCs. This may represent an enhanced Ca^2+^ influx, which can potentiate M-like currents, but the precise nature of this phenomenon remains to be identified.

Our electrophysiological experiments focused on whole-cell net current, so overlapping ionic conductances may have masked the individual current kinetics. Yet, the current recorded during voltage stimulations of PVN MNCs in the presence of RXFP3 agonist frequently resembled the delayed rectifier kinetics characteristic of M-currents, and its involvement was further supported by the dose-dependent pharmacological blockade of RXFP3-induced current by XE991. It should be noted that XE991 does not selectively block M-current/KCNQ channels and may influence other potassium channels (KCNA2 IC_50_ >100 μm, and KCND3 IC_50_ of 43 μm) (Wang et al., 1998). Nonetheless, the concentrations used in the present study were too low to strongly influence KCNA2 or KCND3, which contributes to transient A-currents that would have been largely inactive in our recording protocol, as A-currents become only transiently active on depolarization from a hyperpolarized membrane potential. XE991 has been also shown to block *ether-a-go-go*-related (ERG, KCNH family) K^+^ channels (Wang et al., 1998; Elmedyb et al., 2007), which are present in PVN (Papa et al., 2003) and also mediate M-like currents (Meves et al., 1999; Selyanko et al., 1999; Hirdes et al., 2004). At the same time, XE991 is not a potent ERG blocker (IC_50_ >100 μm); therefore, the efficacy of the concentrations used in the present study (10 and 50 μm) suggests that its effects were mainly due to KCNQ channels' blockade.

In a majority of neurons expressing M-current, the underlying ionic channels are composed of KCNQ2 and KCNQ3 subunits heteromers, and KCNQ2 homomers (Brown and Passmore, 2009). In line with previous reports of KCNQ subunit expression in SON MNCs (W. Zhang et al., 2009), here we observed coexpression of RXFP3 and KNCQ channel subunits in PVN MNCs, with the KCNQ family represented mainly by KCNQ2, which provides a possible molecular substrate for the modulation of the M-current by RLN3/RXFP3 signaling.

Although RXFP3 has been shown to modulate synaptic inputs to parvocellular PVN CRF neurons (C. Zhang et al., 2017) and neurons of the bed nucleus of stria terminalis in mice (Ch'ng et al., 2019), neither RLN3 nor a selective RXFP3 agonist influenced excitatory NMDA/AMPA or inhibitory GABA_A_ receptor-mediated postsynaptic currents (spontaneous and miniature) recorded from PVN MNCs in our rat brain preparations. We therefore conclude that RLN3 inputs primarily act on postsynaptically located RXFP3 to modulate MNC activity in the rat.

We observed no significant sex differences in the influence of RLN3/RXFP3 signaling on MNC electrophysiology. Consequently, the stronger orexigenic effect of central administration of RLN3 in female than in male rats (Calvez et al., 2015, 2016a; Lenglos et al., 2015) might be associated with the overall action of centrally injected peptide at multiple sites, rather than a specific influence on the PVN MNCs. Indeed, central RLN3 administration, leading to increased food intake, has been accompanied by changes (some of them sex-specific) in the expression of several hypothalamic peptide mRNA species as well as increased c-*fos* mRNA in different hypothalamic areas (Calvez et al., 2015, 2016a; Lenglos et al., 2015), reflecting the broad influence of central peptide injection.

Our immunohistochemical and neural tract-tracing studies characterizing the RLN3 innervation of the rat PVN are in line with previous reports of scarce RLN3 fibers within the PVN and a low number of PVN-innervating NI neurons (Olucha-Bordonau et al., 2003; Ma et al., 2007). Similarly, the relative abundance of RLN3 fibers in the peri-PVN zone and the higher number of retrogradely labeled RLN3 neurons in cases involving tracer outside the PVN borders were described in our earlier study in male Wistar rats (Kania et al., 2017). The consistent expression of RXFP3 in the majority of PVN MNCs and their sensitivity to RXFP3 ligands suggest that this innervation pattern may constitute the basis of volume transmission by the peptide that is sufficient to modulate the MNC activity, in addition to canonical synaptic transmission. Sex differences in the RLN3 innervation of PVN, with females displaying a higher density of RLN3-ir fibers, are in line with reports of higher levels of RLN3 mRNA in the female NI (Lenglos et al., 2013). These differences point to higher levels of RLN3 release in female PVN and may contribute to the increased susceptibility of females to BED, in which the RLN3/RXFP3 signaling have been implicated (this study; Lenglos et al., 2013; Calvez et al., 2016b).

Our neuropharmacological studies demonstrated that RXFP3 signaling in the PVN is involved in the BE of HPF in female rats. In line with this observation, intracerebroventricular injection of the RXFP3 antagonist, R3(B1-22)R, reduced HPF intake in rats and mice (Smith et al., 2014; Calvez et al., 2016b). Moreover, earlier reports using different experimental models of BE (Lenglos et al., 2013; Calvez et al., 2016b) have shown that RLN3 expression in the NI and RXFP3 expression in the PVN and SON are elevated in BE female rats, indicating the involvement of RLN3 actions in the nonhomeostatic intake of HPF. The PVN and SON constitute the major source of OXT and AVP, two potent anorexigenic peptides (Meyer et al., 1989; Arletti et al., 1990; Pei et al., 2014; Yoshimura et al., 2017), with OXT being strongly linked to regulation of sweet and palatable food intake (Leng and Sabatier, 2017). Hence, the robust inhibitory action of RXFP3 activation on the activity of OXT and AVP MNCs in PVN documented here and elsewhere (Kania et al., 2017), together with the reduced PVN c-*fos* mRNA levels observed in BE females (Lenglos et al., 2013) and the RXFP3 activation-dependent reduction of OXT and AVP mRNA expression (Ganella et al., 2013a), point to strong inhibition of PVN OXT and AVP signaling during BE episodes. Therefore, future research should aim to determine the specific role of RXFP3-expressing subpopulations of PVN cells (magnocellular vs parvocellular and OXT- vs AVP-synthesizing neurons) in the occurrence and time course of BE behavior.

The PVN is a hub of central homeostatic control, modulating hormonal release and autonomic responses. Current data, together with previous reports (McGowan et al., 2005; Ganella et al., 2013a; Lenglos et al., 2013; Calvez et al., 2015, 2016b; Kania et al., 2017; de Ávila et al., 2018) provide extensive evidence that RLN3/RXFP3 signaling in the PVN constitutes a novel addition to our understanding of the neural circuitry governing physiological food intake, as well as abnormal eating behavior. Moreover, as OXT- and AVP-synthesizing MNCs control fundamental biological processes, including water balance, reproduction, nociception, and a variety of social and parenting behaviors (for review, see Gimpl and Fahrenholz, 2001; Koshimizu et al., 2012), a direct inhibitory action of RLN3 on these neurons has broad behavioral implications. Notably, RLN3-synthesizing neurons are highly stress-responsive (Tanaka et al., 2005; Ma et al., 2013), which together with sex differences in RLN3 actions (for review, see Calvez et al., 2017) and the involvement of RXFP3 in BE behavior modulation shown here, provide a strong rationale for investigating the role of RLN3/RXFP3 signaling in the development of stress-related psychiatric conditions (Kumar et al., 2017), particularly in stress-related eating disorders that differentially affect men and women.
